# Effectiveness and Safety of RHA3 vs a Comparator Product for Lip Augmentation: A Randomized, Controlled, Prospective, Multicenter Clinical Study

**DOI:** 10.1093/asj/sjaf135

**Published:** 2025-07-14

**Authors:** Susan Weinkle, Steven H Dayan, Zoe Draelos, Sabrina Fabi, John Joseph, Joely Kaufman-Janette, Ava Shamban, Bárbara Magalhães, Alice Melotti

## Abstract

**Background:**

Lip augmentation using dermal fillers is increasingly popular, but often requires large volumes and regular touch-ups, whereas poor techniques and product selection can result in unnatural-looking lips. The RHA collection was designed to have less rigidity, allowing the products to adapt to facial animation. In particular, RHA3 has been approved in Europe for lip volumization.

**Objectives:**

The authors of this study aimed to evaluate the effectiveness and safety of RHA3 vs an active comparator for lip augmentation in the US population.

**Methods:**

This was a randomized, controlled, double-blinded, multicenter clinical study. The primary endpoint aimed to demonstrate the noninferiority of RHA3 vs the comparator using the Teoxane Lip Fullness Scale (TLFS), assessed by the blinded live evaluator, 12 weeks after treatment. Secondary objectives included improvement on the TLFS, Global Aesthetic Improvement Scale, patient satisfaction, and natural look/feel of the lips up to 52 weeks. Safety assessment covered adverse events (AEs), common treatment reactions, and injection site pain.

**Results:**

A total of 202 patients were enrolled. RHA3 was considered statistically noninferior to the comparator for lip augmentation among patients with TLFS Grades 1 to 3. It provided sustained lip volume enhancement over time, with high rates of aesthetic improvement and patient satisfaction. Most RHA3-treated patients achieved a natural look and feel of the lips that was maintained throughout the study period. Most AEs were mild to moderate, with no late-onset reactions or angioedema reported.

**Conclusions:**

RHA3 was effective for lip augmentation, providing sustained aesthetic improvement, high satisfaction, and good tolerability. These findings support the use of RHA3 as a uniquely dynamic option for natural-looking lip augmentation.

**Level of Evidence: 1 (Therapeutic):**

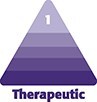

The lips are essential to the aesthetics of the lower face and overall facial balance, with full lips and a well-defined Cupid's bow often associated with youth and beauty.^1-3^ The lips also play an important role in conveying facial emotions and expressions.^2^ Consequently, lip augmentation has become increasingly popular over the past 25 years and, in 2022, lip volumization was among the most requested minimally invasive cosmetic treatments.^4,5^

One of the top priorities for patients seeking lip augmentation is natural-looking results.^6^ Selecting a dermal filler that readily adapts to the movements of native tissue is crucial for achieving natural-looking outcomes in highly mobile areas, specifically the lips.^6,7^ Hyaluronic acid (HA) dermal fillers have different rheological properties, such as strength, cohesivity, and stretch (malleability), depending on HA concentration, molecular weight, the degree of crosslinking, and method used for crosslinking during manufacturing.^7,8^ These properties, as well as the capacity of the HA filler for tissue integration, can impact the outcomes of a procedure.^9^

Several HA dermal fillers have been approved by the US FDA for lip augmentation. However, many of these can result in unnatural-looking stiff lips caused by a poor injection technique, overinjection, or improper choice of fillers, with reports often appearing in the media.^10,11^ Many dermal fillers require large volumes and regular touch-ups to maintain desired outcomes, highlighting an unmet need in the aesthetic medicine market for products that can produce natural-looking and long-lasting effects, with minimal injection volumes and fewer touch-up treatments.^12,13^ Additionally, people with naturally full lips often seek augmentation, but this population is rarely addressed in trials, meaning that the efficacy of dermal fillers in fuller lips requires further validation.

RHA (TEOSYAL Resilient Hyaluronic Acid, Teoxane SA, Geneva, Switzerland; RHA approved in the United States by the FDA) gels are dynamic and cohesive and were specifically designed to adapt to facial animation. The crosslinking reaction conditions of RHA, the Preserved Network Technology, are optimized to preserve high-molecular-weight HA from degradation.^7,14^ This results in longer chains that form a densely entangled network, requiring fewer covalent bonds for stabilization, while maintaining a noninferior clinical persistence.^7,14-16^ The RHA range therefore has a substantially lower degree of modification than other monophasic HA fillers.^7,17^ This leads to decreased rigidity and maintains the ability of HA chains to interact and slide dynamically within the gel.^14^

Among the RHA collection, RHA3 (TEOSYAL RHA 3, RHA Kiss Volume Lido, Teoxane SA; RHA 3 approved in the United States by the FDA) is a viscoelastic, homogeneous, and biodegradable gel crosslinked with 1,4-butanediol diglycidyl ether.^18^ It is indicated for mid-to-deep dermal injection to correct dynamic facial wrinkles and folds, such as the nasolabial folds, in the United States and more recently for lip volumization in Europe.^18^ Its rheologic properties, namely its balance of high strength and dynamic stretch, makes RHA3 a suitable filler to provide both volume and projection in the lips.^7^

To investigate the performance of RHA3 for enhancement of lip fullness in the US population, a randomized, clinical study was conducted to compare the effectiveness and safety of RHA3 vs an active comparator (Restylane-L, Galderma Laboratories, LP, Dallas, TX, approved in the United States by the FDA), indicated for lip augmentation.

## METHODS

### Study Design and Population

The study was a controlled, randomized, double-blinded, between-patient, multicenter prospective clinical study designed to assess the safety and effectiveness of RHA3 vs a comparator product for lip augmentation. The study was conducted between October 2020 and May 2022 across 7 centers in the United States (NCT04540913).

Eligible patients were adults ≥22 years old, seeking lip augmentation. Patients with either a lip fullness grade of 1 to 3 on the Teoxane Lip Fullness Scale (TLFS), seeking at least 1-point correction for upper and/or lower lips, or were Fitzpatrick skin Type V or VI with TLFS grade of 4 or 5, seeking treatment to the vermillion body for upper and/or lower lips were included.^19^ Patients had to abstain from other facial aesthetic procedures/therapies, be able to follow study instructions, complete all required visits, and give their informed consent before participation.

Patients were excluded from the study if they had a known hypersensitivity to lidocaine and/or amide local anesthetic agents, or any component of the devices, or had a history of severe allergy or anaphylactic shock. Patients were also excluded if they had a medical history, ongoing condition, or previous or planned therapies and procedures that could impact their safety or the scientific integrity of the investigation.

A sample size of 200 patients in the overall study population was calculated to be able to detect any adverse event (AE) in the subpopulation of patients with Fitzpatrick skin Types IV to VI with an incidence rate of at least 2.5% (and any AE with an incidence rate of at least 0.5% in the overall population). Thus, at least 200 patients were to be included in the study to maintain sufficient power for safety analysis.

All patients who received at least 1 injection were included in the safety population. The modified intention-to-treat (mITT) population contained all patients who received treatment and had at least 1 postbaseline primary effectiveness observation, excluding those with baseline TLFS grades of 4 and 5. The latter were included for safety analysis only.

Ethics approval was obtained from the IRB Advarra prior to each center's initiation. The primary investigator of each participating site ensured that the study was conducted in compliance with the Declaration of Helsinki and national regulations applicable to Good Clinical Practice. Informed consent adhered to specific country regulatory requirements. Image rights were obtained via informed consent.

### Study Devices and Treatment Protocol

RHA3 is a dermal filler containing 23 mg/mL crosslinked HA, whereas the comparator product is a particulate filler containing 20 mg/mL crosslinked HA. Both fillers contain lidocaine hydrochloride (0.3% w/w) as a local anesthetic agent.

**Figure 1.**
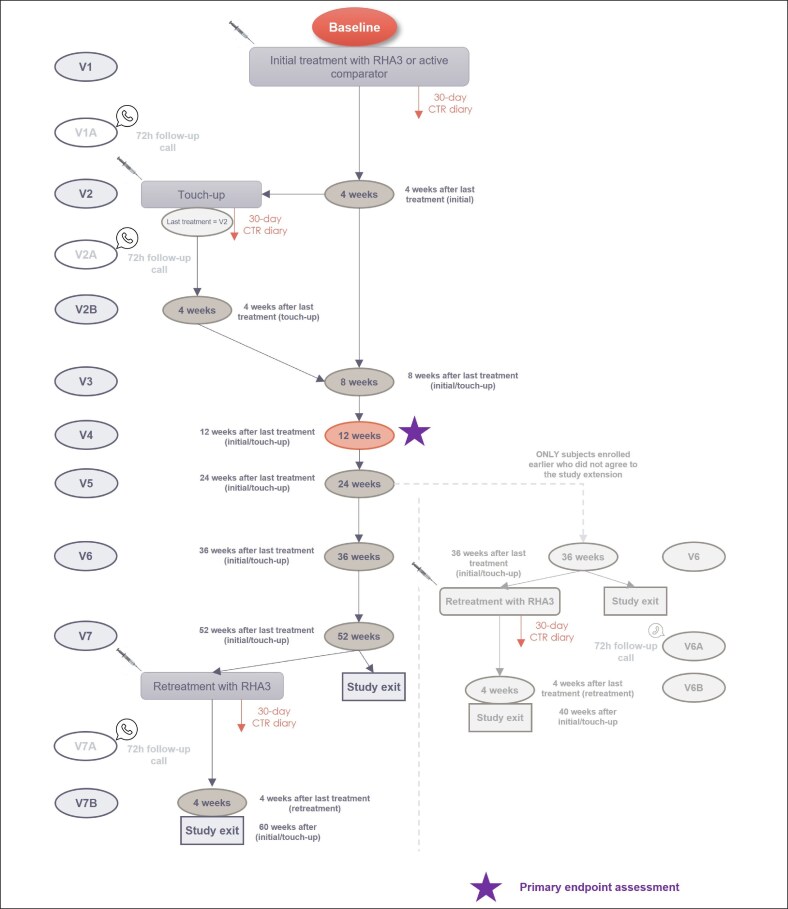
Study design.

Patients were randomized in a 3:1 ratio to receive either RHA3 or a comparator product. The randomization scheme was compliant with the FDA requirements regarding premarket authorization clinical trials for collecting sufficient data on the investigational product in order to demonstrate its safety, performance, and noninferiority toward an FDA-approved comparator. The minimum and maximum anticipated durations of patient participation were 52 and 61 weeks, respectively, for patients who agreed to the extension of their participation, and 36 and 44, respectively, for those who did not agree to the extension of their participation in the study. The investigator was not blinded to the treatment, but the patient and blinded live evaluator (BLE) were. The study design is shown in Figure 1. At Visit 1 (V1), all included patients were injected with the respective device in the vermillion body, vermillion border, and/or oral commissures. An optional touch-up could be performed 4 weeks later (V2), if necessary. A study extension was planned after enrollment had started. Patients who had enrolled before study extension either exited the study 36 weeks after their last treatment (initial or touch-up; V6) or were offered retreatment with RHA3 at V6 and exited 4 weeks later (V6B). Patients who consented to the extended study either exited the study 52 weeks after their last treatment (V7) or were offered retreatment with RHA3 at V7 and exited 4 weeks later (V7B). The final RHA3 retreatments were offered regardless of the patients’ original treatment, if deemed appropriate by the investigator.

Each device was injected into the lip mucosa and/or mid-to-deep dermis as appropriate. The injection technique was chosen at the discretion of the investigator. No more than 3 mL of product were to be administered per visit (1.5 mL per lip). All injections were performed with the 27 G half-inch needle provided with the device.

### Efficacy and Safety Assessments

#### Lip Fullness

Lip fullness was assessed using the 5-grade photonumeric TLFS.^19^ The TLFS score was assessed by the BLE at baseline and subsequently at each visit from 12 weeks after last treatment (V4). The proportion of responders with ≥1-grade improvement on the TLFS was also calculated. The scale was developed and validated through a process similar to those described in the literature for other aesthetic scales, at the request of the FDA.^19-24^ A Clinical Significance Exercise was also conducted on photographs to verify the scale's ability to detect a clinically meaningful difference of 1 grade between patients or before/after treatment. The scale was validated photographically. The scale validation protocol complied with ethical principles of the 1975 Declaration of Helsinki, and informed consent was obtained from each patient participating in this validation process.

#### Satisfaction

Patient satisfaction was assessed using a 5-grade scale at each visit from Week 4 following initial injection (V2) onward. FACE-Q scores were assessed by the patient at each visit from the initial injection visit (V1) for satisfaction with lips and Week 4 (V2) for satisfaction with outcome.^25,26^

#### Global Aesthetic Improvement Scale

The Global Aesthetic Improvement Scale (GAIS) was used to evaluate aesthetic improvement compared with baseline. The GAIS differs slightly between clinical studies, but the version used here scores appearance on a 5-grade scale from “much improved” to “much worse.” GAIS was assessed by the BLE at every visit from Week 12 (V4) and by the treating investigator and patient at every visit from Week 4 (V2).

#### Natural Look and Feel of the Lips

Natural look and feel of the lips were both assessed at each visit using scales from 0 to 10, with 0 being “unnatural” and 10 being “natural.” The proportion of patients reporting a grade ≥7 was calculated.

#### Volume Required to Reach Optimal Cosmetic Result

The volume required to reach optimal cosmetic result (OCR) was the total volume used for initial and touch-up injections of the safety population.

#### Safety

Common treatment reactions (CTRs) were reported by the patient using a diary for 30 days post injection. The severity and duration of each CTR were also reported. CTRs were not classed as AEs unless the severity and/or duration were in excess of that normally observed.

AEs were reported either by the investigator or through the CTR diary, depending on duration. Details of the relationship to the study procedure and device, severity, and seriousness were also recorded.

Injection site pain was assessed using the Visual Analog Scale (VAS) completed by the patient immediately after injection and 5, 15, and 30 min post injection. The VAS is scored from 0 to 100 mm, with 0 representing “no pain” and 100 representing “worst pain.”

### Study Endpoints

The primary efficacy endpoint of the study was the change in the TLFS score from baseline to Week 12 following last injection (V4), as assessed by the BLE. The primary endpoint was met if RHA3 was statistically noninferior to the comparator product. The coprimary endpoint was the proportion of responders with ≥1 grade improvement on the TLFS (TLFS responder rate) at 12 weeks compared with baseline and could only be met for RHA3 if the responder rate was ≥70% for the comparator device.

The secondary efficacy endpoints included the TLFS change from baseline and the TLFS responder rate, both assessed by the BLE from Week 12 (V4) onward. Patient satisfaction, FACE-Q scores, GAIS scores, natural look and feel of the lips, and treatment sessions and volume required to achieve OCR were also assessed.

The exploratory efficacy endpoint was the natural look of the smile as assessed by the patient from weeks following initial injection onward.

The safety endpoints of the study included AEs, CTRs reported in the month following injection, and injection-site pain.

Primary, secondary, and exploratory efficacy analyses were conducted on the mITT population, whereas safety analyses were conducted on the safety population. For the primary efficacy analysis and safety analyses, both RHA3 and comparator product are considered in this work. For secondary and exploratory efficacy analyses, the efficacy of RHA3 was considered vs the comparator product only if deemed pertinent.

### Statistical Analysis

All statistical analyses were performed using SAS software version 9.4 (SAS Institute, Cary). For the primary endpoint, a 2-way analysis of co-variance was used including treatment groups and baseline as a covariate. To determine noninferiority, a 95% 2-sided CI was computed. If the lower bound of the CI was >−0.5, then noninferiority was demonstrated. A post hoc analysis was conducted considering each baseline TLFS grade individually. For TLFS, GAIS, and patient satisfaction, the responder rate was calculated. For FACE-Q, data were Rasch-transformed to a scale of 0 to 100, in which 0 was the worst score and 100 was the best score. For the natural look and feel of the lips, relative frequencies were calculated.

## RESULTS

### Patient Demographics

**Figure 2.**
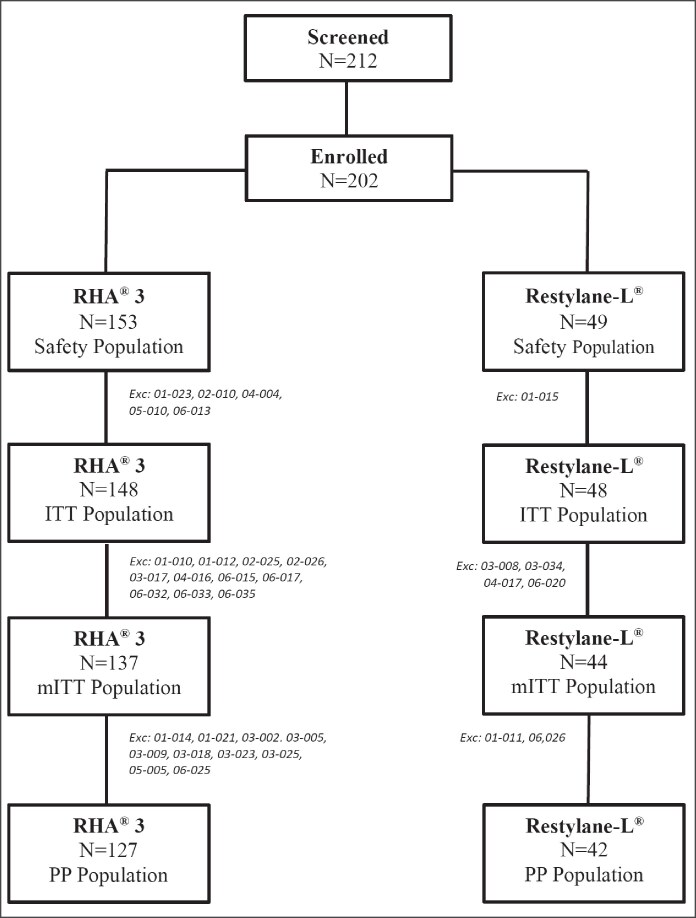
Clinical trial flowchart.

A total of 202 patients were enrolled in the study, all of whom were included in the safety population, and 181 in the mITT population. In total, 111 completed the study with the extension and 74 without the extension (Figure 2).

Of the 202 patients included in the safety population, 199 (98.5%) were female and 3 were male (1.5%) (Table 1). The mean age at inclusion was 48.7 ± 12.8 years. Most patients were White (86.6%), and Type III (38.1%) or Type II (27.2%) on the Fitzpatrick scale, whereas 26.2% were Types IV to VI. Among patients with Fitzpatrick Type V, 62% had a TLFS baseline grade of 4. For those with Fitzpatrick Type VI, the majority had a TLFS baseline grade of either 4 (38%) or 5 (38%). Of the 181 patients in the mITT population, most had a TLFS baseline grade of 3 (47.5%) or 2 (45.9%).

**Table 1.** Demographics of the Safety Population

Variable	RHA3	Comparator	Total
*n*	153	49	202
Age (mean ± SD)	48.8 ± 13.2	48.5 ± 11.7	48.7 ± 12.8
Sex			
Female	151 (98.7%)	48 (98.0%)	199 (98.5%)
Male	2 (1.3%)	1 (2.0%)	3 (1.5%)
Fitzpatrick skin type			
I	10 (6.5%)	7 (14.3%)	17 (8.4%)
II	46 (30.1%)	9 (18.4%)	55 (27.2%)
III	58 (37.9%)	19 (38.8%)	77 (38.1%)
IV	22 (14.4%)	10 (20.4%)	32 (15.8%)
V	10 (6.5%)	3 (6.1%)	13 (6.4%)
VI	7 (4.6%)	1 (2.0%)	8 (4.0%)
Ethnicity			
Hispanic or Latino	32 (20.9%)	13 (26.5%)	45 (22.3%)
Non-Hispanic or Latino	118 (77.1%)	35 (71.4%)	153 (75.7%)
Not available	3 (2.0%)	1 (2.0%)	4 (2.0%)
Race			
White	130 (85.0%)	45 (91.8%)	175 (86.6%)
Black or African American	15 (9.8%)	2 (4.1%)	17 (8.4%)
Asian	4 (2.6%)	1 (2.0%)	5 (2.5%)
American Indian or Alaska native	2 (1.3%)	1 (2.0%)	3 (1.5%)
Native Hawaiian or other Pacific islander	2 (1.3%)	0 (0.0%)	2 (1.0%)

Percentages may not add to 100% due to rounding. For race, percentages may not add to 100% because a patient could be included in more than 1 category.

*n*, number of patients; SD, standard deviation.

### Primary Efficacy Analysis

The mean change in the TLFS score between baseline and Week 12 (V4) was 1.0 ± 0.7 in the RHA3 group and 0.8 ± 0.7 in the comparator group (Table 2). There was no significant difference between the 2 groups. As the lower bound of the 95% CI was above the predetermined noninferiority margin of −0.5, noninferiority was demonstrated for the primary endpoint.

**Table 2.** Primary Efficacy Endpoint and Post hoc Analysis

Variable	RHA3	Comparator
Baseline TLFS Grades 1-3		
*n*	137	44
TLFS change from baseline		
Mean ± SD	1.0 ± 0.7	0.8 ± 0.7
95% CI	(0.9-1.1)	(0.6-1.0)
Min to max	0 to 3	−1 to 2
Responder rate, *n* (%)	107 (78.1%)	29 (65.9%)
Baseline TLFS Grades 1 and 2 only		
*n*	68	27
TLFS change from baseline		
Mean ± SD	1.3 ± 0.6	1.1 ± 0.6
95% CI	(1.1-1.4)	(0.9-1.3)
Min to max	0 to 3	0 to 2
Responder rate, *n* (%)	64 (94.1%)	24 (88.9%)
Baseline TLFS Grade 3 only		
*n*	69	17
TLFS change from baseline		
Mean ± SD	0.7 ± 0.5	0.3 ± 0.6
95% CI	(0.5-0.8)	(0.0-0.5)
Min to max	0 to 2	−1 to 1
Responder rate, *n* (%)	43 (62.3%)	5 (29.4%)

*n*, number of patients; SD, standard deviation; TLFS, Teoxane Lip Fullness Scale.

The proportion of patients with ≥1-grade improvement on the TLFS was 78.1% for the RHA3 group and 65.9% for the comparator group. To meet the coprimary endpoint, the percentage of patients in the comparator group with ≥1-grade improvement needed to be ≥70%. Because this was not the case, the coprimary endpoint was unable to be met.

A literature review of similar studies revealed that the historical efficacy of the comparator product was not assessed in patients with moderately full lips.^27-35^ The difference in inclusion criteria between these studies and the current work made it more challenging for the comparator product to demonstrate at least a 1-point improvement on the TLFS in our study. Thus, a post hoc analysis was conducted on primary and coprimary endpoints in the mITT population to determine the TLFS responder rate of patients with TLFS Grade 3 only and with Grades 1 and 2 at baseline. When considering only patients with TLFS Grades 1 and 2 at baseline, responder rates were 94.1% in the RHA3 group and 88.9% in the comparator group, and RHA3 remained noninferior to the comparator (Table 2). For those with only TLFS Grade 3 at baseline, the responder rate was high for RHA3 (62.3%) but lower for the comparator (29.4%). Therefore, when excluding patients with TLFS Grade 3 at baseline, both the primary and the co-primary endpoints were met. Nevertheless, RHA3 efficacy was demonstrated regardless of the TLFS grade.

### Secondary and Exploratory Efficacy Analyses

#### TLFS Responder Rate

**Figure 3.**
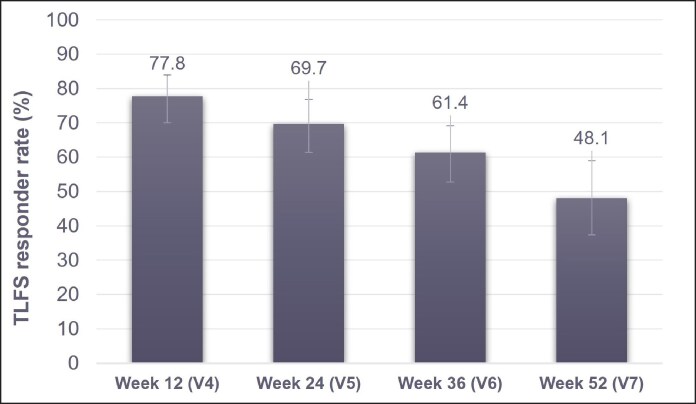
TLFS responder rate after RHA3 treatment, as assessed by the BLE throughout the study period. BLE, blinded live evaluator; TLFS, Teoxane Lip Fullness Scale.

Following RHA3 treatment, the clinically significant TLFS responder rate (≥1-grade improvement) as assessed by the BLE remained at 48.1% by 52 weeks post treatment (V7) (Figure 3). The TLFS responder rate for the comparator group was generally lower than the RHA3 group, including at Week 52 (26.1%, data not available).

#### GAIS Improvement

**Figure 4.**
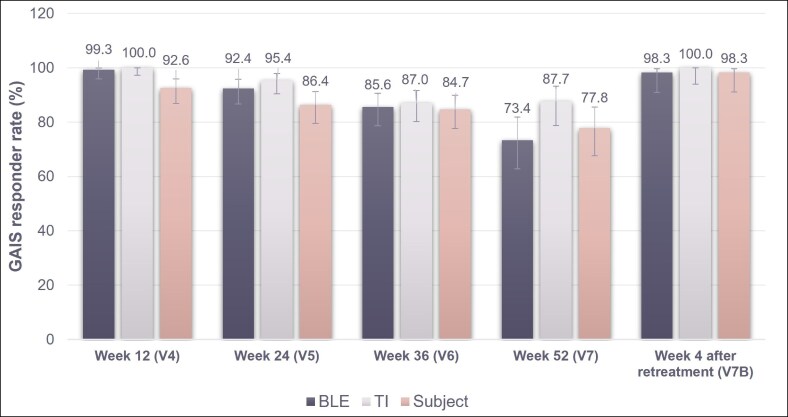
Proportion of patients deemed “improved” or “much improved” on the GAIS assessment after RHA3 treatment throughout the study period, as assessed by the BLE, TI, and patient. BLE, blinded live evaluator; GAIS, Global Aesthetic Improvement Scale; TI, treating investigator.

At Week 12 (V4), the proportion of patients deemed “improved” or “much improved” by the BLE on the GAIS scale (responder rate) was 99.3% (Figure 4). GAIS improvement rates in the RHA3 group were maintained over 73% and significantly higher (*P* < .05) than that of the comparator group at all visits after Week 12 (data not available).

The GAIS responder rate as determined by the patient was 97.1% at 4 weeks after initial RHA3 injection (V2). This increased to 100.0% by Week 4 following touch-up injection (V2B) and remained at least 77.8% after Week 12 (V4).

#### Treatment Sessions and Volume

Initial treatment was defined as the first treatment received by the patient. The mean volume (±SD) injected for initial treatment was 1.43 ± 0.5 mL for RHA3 (upper lip: 0.70 ± 0.3 mL; lower lip: 0.74 ± 0.3 mL) and 1.43 ± 0.5 mL for comparator (upper lip: 0.72 ± 0.3 mL; lower lip: 0.70 ± 0.3 mL).

Touch-up was defined as a treatment performed 4 weeks after initial injection to reach OCR. Touch-up treatments were less frequent in the RHA3 group, with 58.2% of patients in the RHA3 group and 73.5% in the comparator group needing more treatments to achieve optimal outcomes at Week 4 following initial injection (Table 3). The mean volume (±SD) injected for touch-up treatment was 0.60 ± 0.4 mL for RHA3 (upper lip: 0.26 ± 0.2 mL; lower lip: 0.35 ± 0.3 mL) and 0.71 ± 0.4 mL for comparator (upper lip: 0.37 ± 0.2 mL; lower lip: 0.35 ± 0.3 mL).

**Table 3.** Number of Treatment Sessions and Treatment Volume

Variable	RHA3 (*n* = 153)	Comparator (*n* = 49)
Patients receiving treatment		
Initial	153 (100.0%)	49 (100.0%)
Touch-up	89 (58.2%)	36 (73.5%)
Retreatment	90 (58.8%)	30 (61.2%)
Overall injection volume (mean ± SD)		
Both lips	2.39 ± 1.0 mL	2.58 ± 1.0 mL
Upper lip	1.16 ± 0.5 mL	1.31 ± 0.5 mL
Lower lip	1.23 ± 0.6 mL	1.27 ± 0.7 mL
Initial injection volume (mean ± SD)		
Both lips	1.43 ± 0.5 mL	1.43 ± 0.5 mL
Upper lip	0.70 ± 0.3 mL	0.72 ± 0.3 mL
Lower lip	0.74 ± 0.3 mL	0.70 ± 0.3 mL
Touch-up injection volume (mean ± SD)		
Both lips	0.60 ± 0.4 mL	0.71 ± 0.4 mL
Upper lip	0.26 ± 0.2 mL	0.37 ± 0.2 mL
Lower lip	0.35 ± 0.3 mL	0.35 ± 0.3 mL
Retreatment injection volume (mean ± SD)		
Both lips	1.03 ± 0.5 mL	1.03 ± 0.4 mL
Upper lip	0.53 ± 0.3 mL	0.51 ± 0.2 mL
Lower lip	0.50 ± 0.3 mL	0.52 ± 0.3 mL
Injection volume to reach OCR (mean ± SD)		
Both lips	1.78 ± 0.6 mL	1.95 ± 0.7 mL
Upper lip	0.85 ± 0.3 mL	1.00 ± 0.4 mL
Lower lip	0.94 ± 0.4 mL	0.96 ± 0.5 mL

*n*, number of patients; OCR, optimal cosmetic result; SD, standard deviation.

Retreatment was defined as a treatment performed several months after initial or touch-up treatments to correct volume loss. Retreatment was given to 58.2% and 61.2% of patients in the RHA3 and comparator groups, respectively, at 36 or 52 weeks following last injection. The mean volume (±SD) injected for retreatment was 1.03 ± 0.5 mL for RHA3 (upper lip: 0.53 ± 0.3 mL; lower lip: 0.50 ± 0.3 mL) and 1.03 ± 0.4 mL for comparator (upper lip: 0.53 ± 0.2 mL; lower lip: 0.51 ± 0.3 mL).

The overall injection volume comprised the total volume used for initial, touch-up and retreatment injections. The volume of RHA3 injected overall was slightly lower than the comparator (RHA3: 2.39 ± 1.0 mL for both lips [upper lip: 1.16 ± 0.5 mL; lower lip: 1.23 ± 0.6 mL] vs 2.58 ± 1.0 mL for both lips [upper lip: 1.31 ± 0.5 mL; lower lip: 1.27 ± 0.7 mL] for the comparator).

The volume used to achieve OCR was the sum of initial and touch-up (if applicable) injections. The volume of RHA3 injected to achieve OCR totaled 1.78 ± 0.6 mL for both lips (upper lip: 0.85 ± 0.3 mL; lower lip: 0.94 ± 0.4 mL). The volume of comparator product injected to achieve OCR was slightly higher at 1.95 ± 0.7 mL for both lips (upper lip: 1.0 ± 0.4 mL; lower lip: 0.96 ± 0.5 mL).

#### Patient Satisfaction

Patient satisfaction scores were maintained above 79.5% throughout the study following RHA3 treatment (Supplemental Figure 1). Patients in the RHA3 group were more satisfied than those in the comparator group at Week 52 (V7) (data not available).

**Figure 5.**
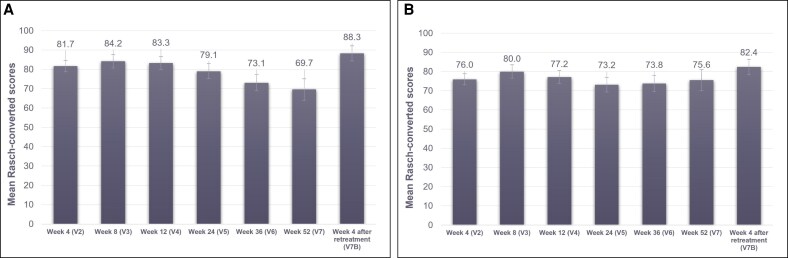
FACE-Q satisfaction after RHA3 treatment throughout the study period. (A) FACE-Q satisfaction with lips; (B) FACE-Q satisfaction with outcomes. Rasch-converted scores ranged from 0 to 100, with higher scores reflecting a positive outcome.

FACE-Q satisfaction with lips increased from 32.5 ± 18.9 at baseline to 81.7 ± 17.2 at Week 4 (V2) in the RHA3 group (Figure 5A). FACE-Q satisfaction with lips remained high throughout the study with a score of 69.7 ± 25.1 at Week 52.

FACE-Q satisfaction with outcome was the highest at Week 8 (V3) (80.0 ± 22.6) but remained above 73.2 throughout the study with a score of 75.6 ± 24.1 at Week 52 (Figure 5B).

#### Naturalness of Lips and Smile

**Figure 6.**
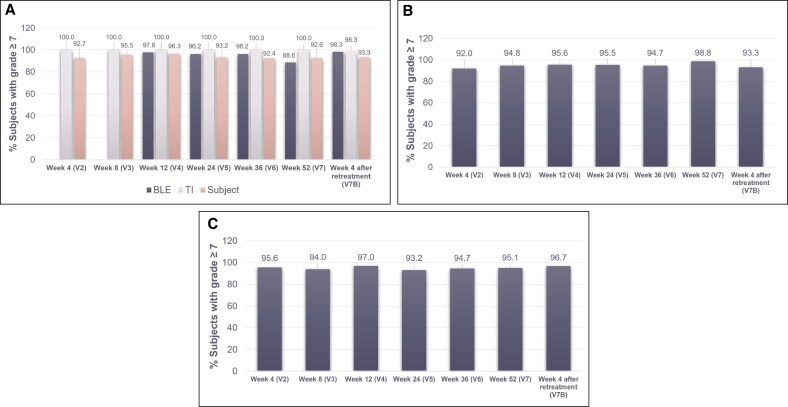
Naturalness of treatment. (A) Natural look of lips over time as assessed by the BLE, TI, and patient. (B) Natural feel over time as assessed by the patient. (C) Natural look of smile over time as assessed by the patient. BLE, blinded live evaluator; TI, treating investigator.

When assessed by the BLE, at least 88.6% of patients had natural-looking lips throughout the study in the RHA3 group (Figure 6A). This was the lowest at Week 52 (V7) and increased to 98.3% following retreatment. Similarly, when assessed by the patient, at least 92.4% of patients treated with RHA3 deemed their lips natural looking throughout the study, with consistent results across all visits.

Based on patient assessment, at least 92.0% of patients treated with RHA3 had a natural feel of the lips throughout the study (Figure 6B).

With regard to the natural look of the smile, at least 93.2% of patients treated with RHA3 considered their smile natural looking throughout the study (Figure 6C).

**Figure 7.**
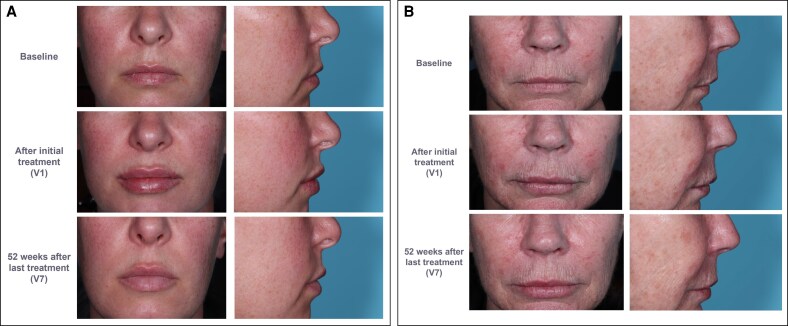
Before and after photographs of (A) a 42-year-old female patient and (B) a 63-year-old female patient who participated in the study, from a frontal and profile view, at baseline, immediately after initial treatment (V1), and 1 year after last treatment (initial or touch-up; V7).

Representative pretreatment, immediately after initial treatment (V1), and 52 weeks after last treatment (V7) images of 4 patients are shown in Figure 7 and Supplemental Figure 2.

### Safety Analysis

Across both treatment arms, a total of 277 AEs were reported by 97 patients (48.0%); 153 across 75 patients (49.0%) in the RHA3 group and 49 across 22 patients (44.9%) in the comparator group. Of these, 194 AEs across 78 patients (38.6%) were considered related to the study device; 144 across 63 patients (41.2%) in the RHA3 group and 50 across 15 patients (30.6%) in the comparator group. All adverse device effects (ADEs; AE related to the use of an investigational medical device or comparator) in both treatment groups were either mild or moderate. The most common ADEs were mass (RHA3: 14.4%; comparator: 16.3%), swelling (RHA3: 9.2%; comparator: 12.2%) or induration (RHA3: 5.2%; comparator: 6.1%) at the injection site. There were no cases of angioedema and no delayed onset reactions.

Just 1 AE of special interest was reported in the RHA3 group: a case of blurred vision that was considered unlikely to be related to the study treatment or procedure. One serious AE was reported in the comparator group: a case of intracranial aneurysm, which was considered unrelated to the device.

Following treatment with RHA3, most patients (95.2%) reported at least 1 CTR following initial injection. The most common CTRs following an initial injection of RHA3 were swelling, firmness, lumps/bumps, and tenderness, all reported in above 75% of patients (Table 4). The percentage of patients in the RHA3 group experiencing a CTR was lower following touch-up (81.8%) and retreatment (87.5%) injections. The most reported CTRs remained as swelling, firmness, lumps/bumps, and tenderness, reported in above 50% of patients following touch-up and above 60% of patients following retreatment. CTRs were most commonly mild or moderate and resolved in 1 to 3 days, regardless of treatment session. The rate of CTRs was similar between groups; however, pain was reported less frequently in the RHA3 group following initial (52.4% vs 64.6%) and touch-up (30.7% vs 48.5%) injections. Following touch-up injection, redness (29.5% vs 45.5%) and discoloration (15.9% vs 27.3%) were also reported less frequently in the RHA3 group than comparator.

**Table 4:** Common Treatment Reactions Reported By Patients in the 30 Days Following Injection

Injection	Reaction	RHA3	Comparator	Total
Initial	*n*	147	48	195
	Any CTR	140 (95.2%)	47 (97.9%)	187 (95.9%)
	Redness	81 (55.1%)	28 (58.3%)	109 (55.9%)
	Pain	77 (52.4%)	31 (64.6%)	108 (55.4%)
	Tenderness	114 (77.6%)	38 (79.2%)	152 (77.9%)
	Firmness	115 (78.2%)	38 (79.2%)	153 (78.5%)
	Swelling	134 (91.2%)	47 (97.9%)	181 (92.8%)
	Lumps/bumps	115 (78.2%)	38 (79.2%)	153 (78.5%)
	Bruising	102 (69.4%)	25 (52.1%)	127 (65.1%)
	Itching	39 (26.5%)	9 (18.8%)	48 (24.6%)
	Discoloration	65 (44.2%)	20 (41.7%)	85 (43.6%)
Touch-up	*n*	88	33	121
	Any CTR	72 (81.8%)	26 (78.8%)	98 (81.0%)
	Redness	26 (29.5%)	15 (45.5%)	41 (33.9%)
	Pain	27 (30.7%)	16 (48.5%)	43 (35.5%)
	Tenderness	49 (55.7%)	18 (54.5%)	67 (55.4%)
	Firmness	51 (58.0%)	19 (57.6%)	70 (57.9%)
	Swelling	55 (62.5%)	25 (75.8%)	80 (66.1%)
	Lumps/bumps	50 (56.8%)	21 (63.6%)	71 (58.7%)
	Bruising	28 (31.8%)	13 (39.4%)	41 (33.9%)
	Itching	12 (13.6%)	5 (15.2%)	17 (14.0%)
	Discoloration	14 (15.9%)	9 (27.3%)	23 (19.0%)
Retreatment	*n*	88	26	114
	Any CTR	77 (87.5%)	20 (76.9%)	97 (85.1%)
	Redness	45 (51.1%)	14 (53.8%)	59 (51.8%)
	Pain	44 (50.0%)	12 (46.2%)	56 (49.1%)
	Tenderness	65 (73.9%)	18 (69.2%)	83 (72.8%)
	Firmness	66 (75.0%)	18 (69.2%)	84 (73.7%)
	Swelling	70 (79.5%)	20 (76.9%)	90 (78.9%)
	Lumps/bumps	61 (69.3%)	16 (61.5%)	77 (67.5%)
	Bruising	57 (64.8%)	15 (57.7%)	72 (63.2%)
	Itching	17 (19.3%)	5 (19.2%)	22 (19.3%)
	Discoloration	37 (42.0%)	10 (38.5%)	47 (41.2%)

CTR, common treatment reaction; *n*, number of patients.

Injection site pain as measured by the VAS was mild to moderate for both treatment groups immediately following initial (9.0 ± 14.7 mm), touch-up (8.3 ± 17.7 mm), and retreatment injections (Week 36 [V6]: 7.0 ± 16.7 mm; Week 52 [V7]: 2.7 ± 7.9 mm). Pain immediately decreased, with mean VAS scores approaching zero by just 5 min post injection. Mean VAS scores were similar between RHA3 and comparator at all visits.

## DISCUSSION

Lip augmentation is one of the most sought-after minimally invasive cosmetic procedures, but often requires large volumes of dermal filler and can result in a unnatural-looking lips.^4^ RHA dermal fillers were designed to adapt to facial animation. In particular, the balance of high strength and dynamic stretch of RHA3 makes it ideal for adding volume and projection to the lips, and it is indicated for this purpose in ex-US countries.^7,18^ The authors of this study have demonstrated that RHA3 is statistically noninferior to an active comparator product for lip augmentation in patients with thin to moderately full lips (TLFS Grades 1-3). Notably, the comparator product showed a lack of efficacy in individuals with TLFS Grade 3 at baseline, whereas RHA3 exhibited a good performance in this subset of patients. RHA3 provided sustained lip augmentation over time with TLFS responder rates that were generally higher than those of the comparator group. RHA3 also provided sustained improvement on the GAIS scale, with significantly higher GAIS responder rates (as assessed by the BLE and patient) than the comparator group at between 24 and 52 weeks following the last treatment. Injected volumes corresponded to those needed to achieve OCR according to the injector and as assessed by the BLE after initial treatment and/or touch-up when applicable. Mean volumes were similar between both the RHA3 and the comparator groups and remained below the maximum authorized volumes of 3 mL for the comparator. RHA3 treatment produced high patient satisfaction, with higher satisfaction at 1-year post treatment than the active comparator. Most patients treated with RHA3 achieved a natural look and feel of the lips that was maintained throughout the study period. Despite the use of similar initial volumes between groups, the RHA3 group required a numerically lower volume and fewer treatments to achieve similar or higher efficacy, as well as similar safety. RHA3 exhibited a favorable safety profile, including no late-onset reactions and no angioedema, which are both possible, although rare, following the injection of the dermal filler.^36-40^

The authors of many studies evaluating the efficacy of HA dermal fillers for lip augmentation primarily focus on patients with thin lips.^27-35^ When patients with moderately full lips are included, the sample size is often small and data are combined with that of patients with thinner lips.^41,42^ Consequently, the effectiveness of HA dermal fillers for lip augmentation in individuals with moderately full lips has been relatively underexplored. The authors of this study are therefore one of the first to demonstrate the long-term effectiveness of lip augmentation in individuals with moderately full lips before treatment.

The current study is one of the few lip augmentation studies to have followed patients for up to 52 weeks following treatment.^43^ The authors of other studies who have followed patients for this length of time found similar or greater decreases in responder rates on the GAIS or the relevant lip fullness scale by 12 months after treatment.^30,41,42^ The authors of this study have therefore demonstrated that RHA3 has long-term benefits in terms of aesthetic improvement and patient satisfaction with lip augmentation. The long durability of RHA3 may be due to its crosslinking technology combined with the preservation of high-molecular-weight HA chains that allows for lower rigidity and, therefore, improves adaptivity to dynamic areas such as the lips.^7^ Collectively, these properties of RHA3 are likely to contribute to its longevity and long-lasting benefits.

One of the challenges of this analysis was that the coprimary endpoint required the comparator product to have a responder rate of at least 70%. The coprimary endpoint could not be achieved due to the comparator's insufficient responder rate at Week 12. This led to a closer examination of the inclusion criteria, in which we identified that similar studies often only include patients with very thin to thin lips at baseline, and therefore the efficacy of the comparator product was not established in patients with moderately full lips.^27-35^ Post hoc analysis confirmed that, although the responder rate was high in RHA3-treated patients with fuller lips at baseline, those treated with the comparator product had a low responder rate. In the future, more robustly powered studies should be conducted to understand the efficacy and safety of HA dermal fillers in patients with fuller lips.

Despite almost a third of US cosmetic procedures being performed on non-Caucasians in 2019, authors of very few clinical studies include patients with darker skin types.^44,45^ Of the limited studies that exist in people of color (POC), dermal fillers have exhibited good safety profiles in this patient population, including HA gels from the RHA collection.^23,45-49^ However, the safety of dermal fillers in POC for lip augmentation has not yet been determined. In our study, over 1 quarter of the patients included in the safety population were Fitzpatrick skin Types IV to VI. Although RHA3 was well-tolerated across all patients, a future post hoc analysis could compare the safety of Fitzpatrick skin Types IV to VI with Types I to III to rule out any safety concerns surrounding lip augmentation with RHA3. Large-scale future studies could also be conducted to compare the safety and efficacy of RHA3 in POC.

The study is limited in its ability to compare results with other studies because of the proprietary nature of the TLFS and the scales used in other studies.^50,51^ However, all investigators and BLEs were certified in using the scale, meaning that the results throughout the study can be considered consistent, even though they cannot be compared with those from studies using other scales. Despite the fact that the investigators were not blinded in the context of this study and which could present a bias, the inclusion of the assessment performed by the BLE following initial treatment and/or touch-up allowed to mitigate such bias.

The results from this study support the use of RHA3 as a competitive option for lip augmentation in different TLFS grades (1-3) and in a diversified population. In addition to lip augmentation, the authors of this study demonstrated that RHA3 is versatile enough to be used to treat the oral commissures, making RHA3 the only product approved for lip augmentation with oral commissures as an indicated area of treatment.^18^ Therefore, RHA3 may be beneficial in individuals seeking a comprehensive lip augmentation beyond just the vermillion body.

Given the versatile rheological properties and dynamic applications of the RHA collection, RHA3 could be combined with other RHA products to optimize enhancement of the lips, as well as of the perioral area. For example, RHA Redensity (or RHA1 in Europe) benefits from a very high stretch and is suited for the correction of superficial, dynamic wrinkles in the perioral region, such as perioral rhytids, lips including philtrum, and oral commissures; RHA2 is a gel with high stretch and moderate strength, designed to correct moderate to severe dynamic wrinkles, such as nasolabial folds, while also augmenting the volume of facial tissues in the perioral area (ie, perioral rhytids, lips including philtrum, marionette lines, oral commissures); the dynamic volumizer RHA4 has very high strength and moderate stretch, making it ideal for the correction of deep, dynamic facial wrinkles, such as nasolabial folds.^7^ It is also used for facial contour remodeling and volume restoration/augmentation in the perioral region including marionette lines.^52-56^ These products could therefore be layered to address the lips and any wrinkles or creases in the perioral area, to achieve optimal outcomes. Additionally, future studies could evaluate RHA3 in combination with other RHA fillers to obtain comprehensive outcomes in the lower face.

## CONCLUSIONS

The authors of this study have demonstrated the safety and effectiveness of RHA3 for lip volumization in individuals with thin to moderately full lips. RHA3 was noninferior to the active comparator, providing sustained lip volume enhancement over time with high rates of aesthetic improvement and patient satisfaction. The effectiveness trended toward being greater than the comparator product in individuals with fuller lips at baseline. The natural look and feel of the lips were maintained throughout the study period, whereas numerically lower volumes and fewer touch-ups were required vs the comparator product. RHA3 also exhibited a favorable safety profile, including no late-onset reactions or angioedema. Ultimately, RHA3 is a strong competitive option for lip augmentation across various TLFS grades (1-3) within the US population.

## Supplementary Material

sjaf135_Supplementary_Data
