# Supplementary material for: Effectiveness and Safety of RHA3 vs a Comparator Product for Lip Augmentation: A Randomized, Controlled, Prospective, Multicenter Clinical Study
Source: Aesthet Surg J. 2025 Jul 14;45(11):1175–86. doi: 10.1093/asj/sjaf135 (PMC12529664; doi:10.1093/asj/sjaf135)
Supplement: sjaf135_Supplementary_Data [file sjaf135_supplementary_data.zip › Supplemental Figure Legend.docx]

**Supplemental Figure Legend**

**Supplemental Figure 1.** Subject satisfaction scores after RHA3 treatment throughout the study period.

**Supplemental Figure 2.** Before and after photographs of a (A) 56-year-old female subject and a (B) 46-year-old female subject who participated in the study, from a frontal and profile view, at baseline, immediately after initial treatment (V1), and 1 year after last treatment (initial or touch-up; V7).
