# Supplementary figures and images for: Effectiveness and Safety of RHA3 vs a Comparator Product for Lip Augmentation: A Randomized, Controlled, Prospective, Multicenter Clinical Study
Source: Aesthet Surg J. 2025 Jul 14;45(11):1175–86. doi: 10.1093/asj/sjaf135 (PMC12529664; doi:10.1093/asj/sjaf135)

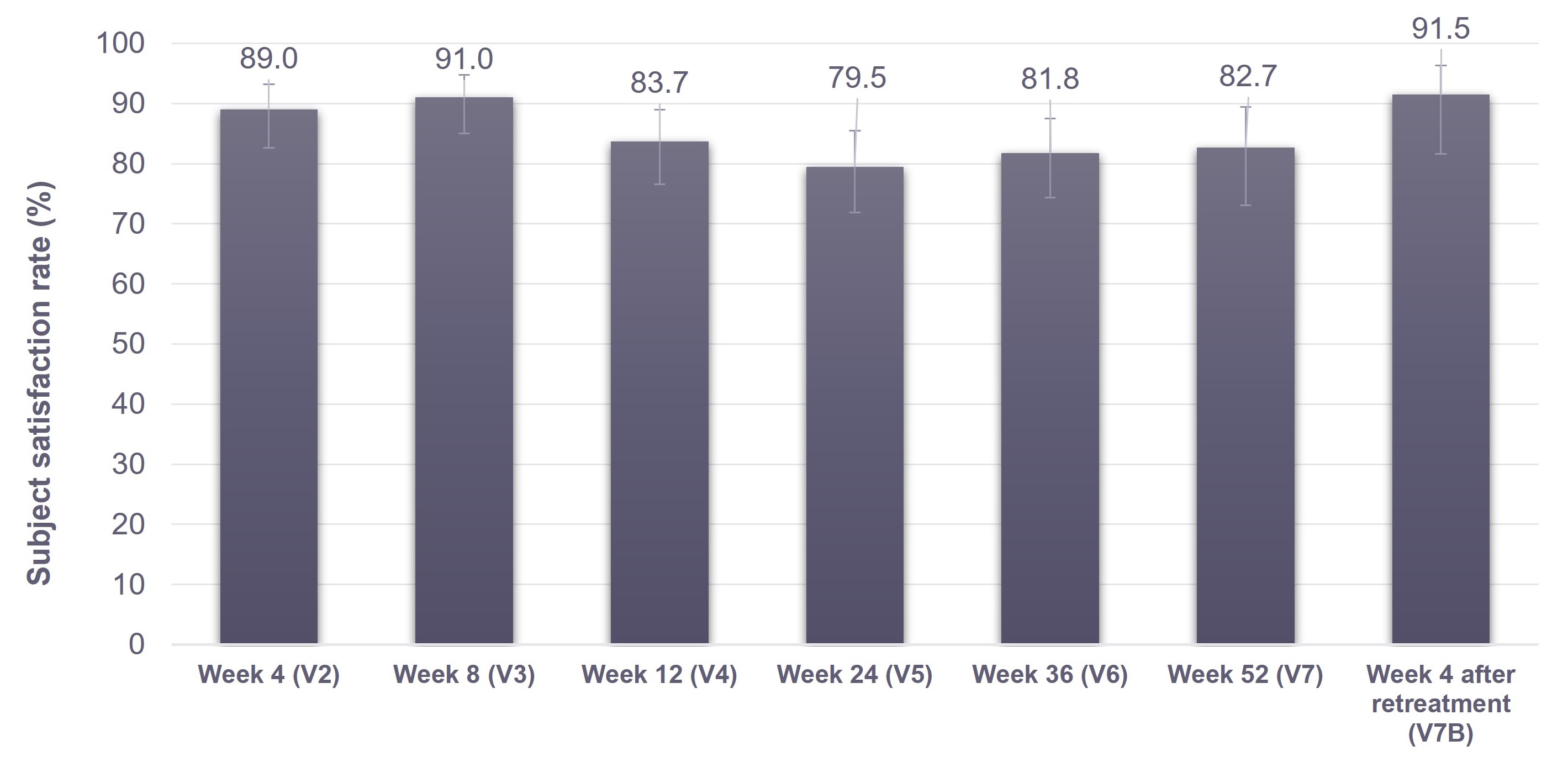

Supplement: sjaf135_Supplementary_Data [file sjaf135_supplementary_data.zip › Supplemental Figure 1.jpg]

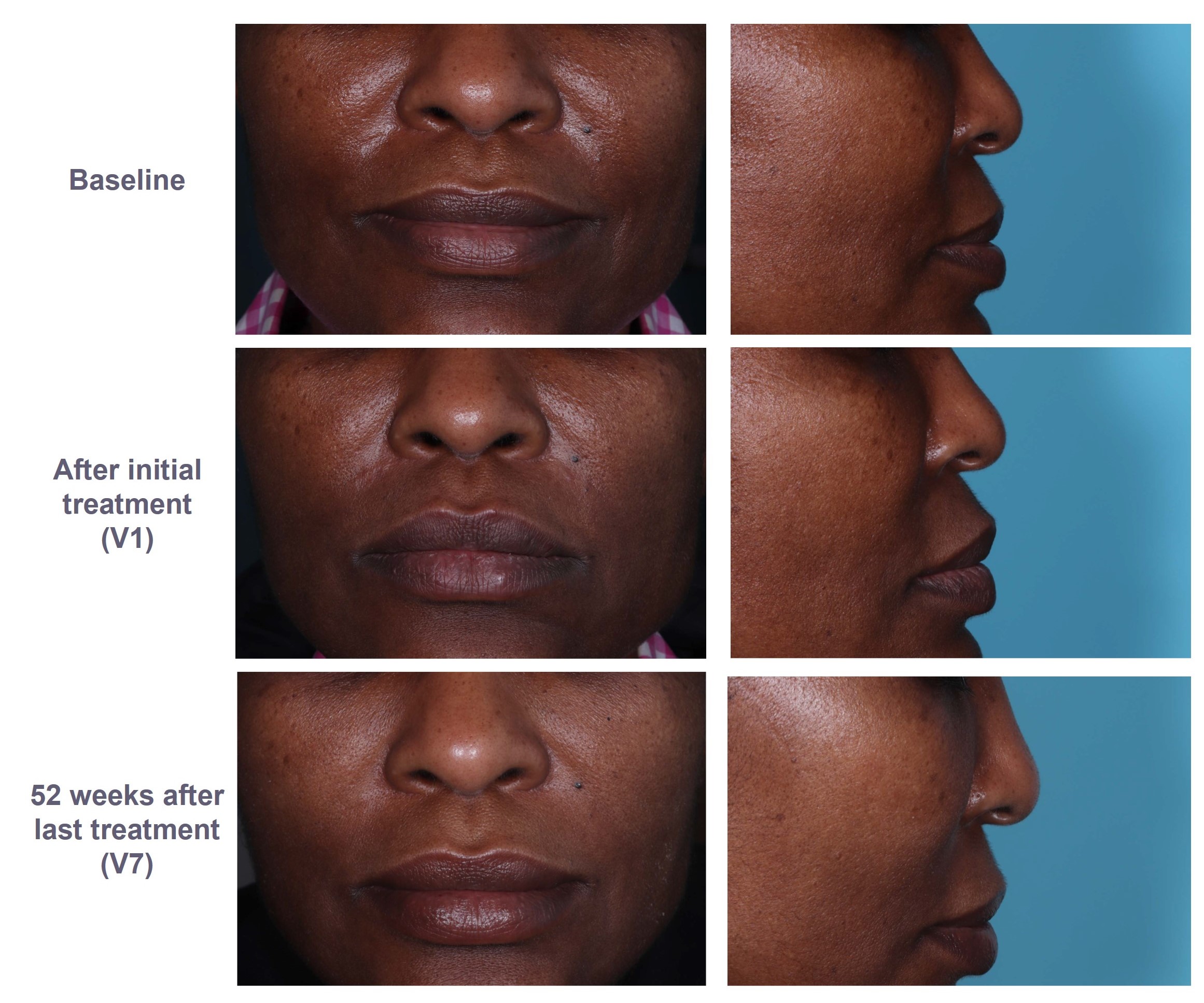

Supplement: sjaf135_Supplementary_Data [file sjaf135_supplementary_data.zip › Supplemental Figure 2A.jpg]

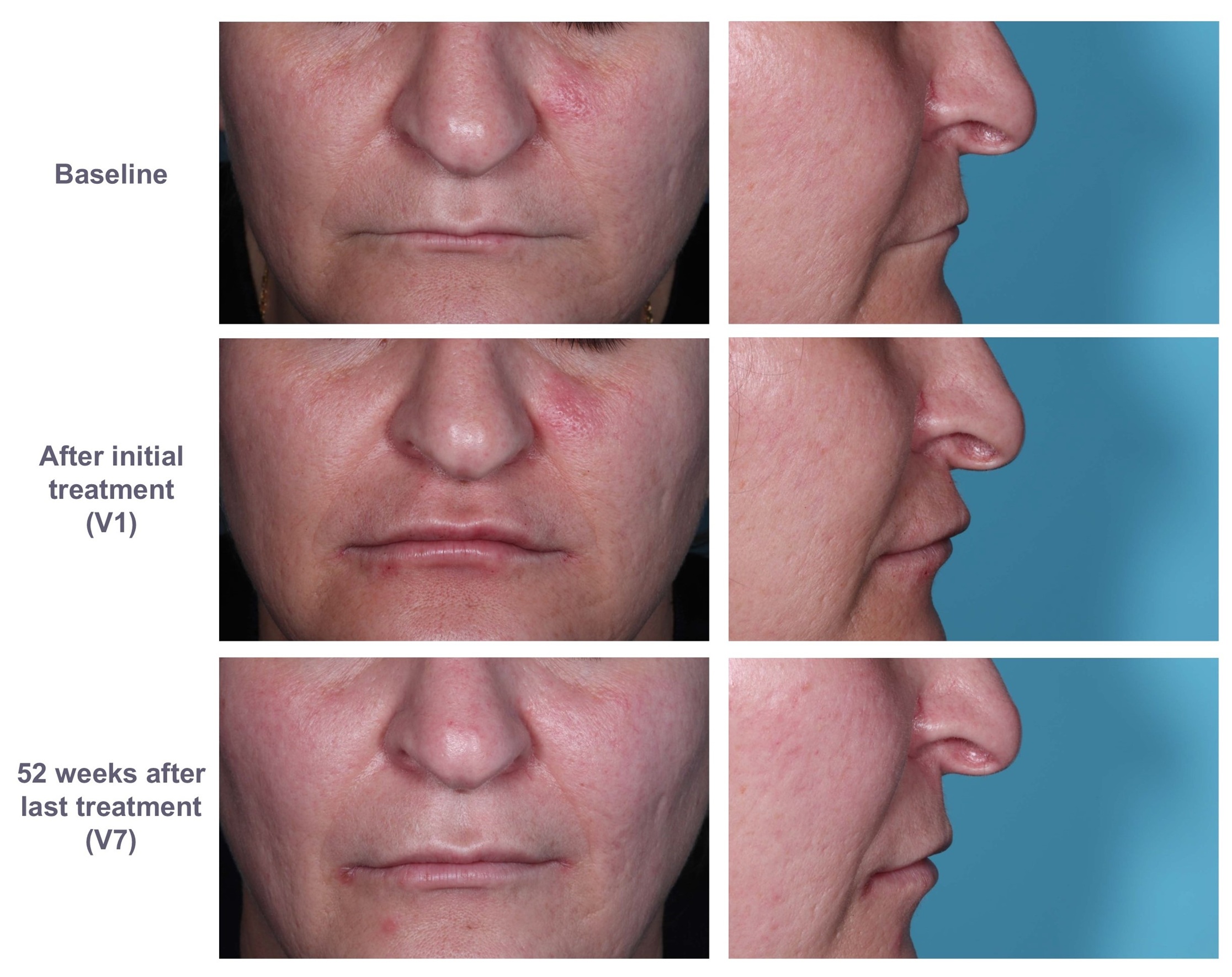

Supplement: sjaf135_Supplementary_Data [file sjaf135_supplementary_data.zip › Supplemental Figure 2B.JPG]
